# Roles of the vestibular system in obesity and impaired glucose metabolism in high-fat diet-fed mice

**DOI:** 10.1371/journal.pone.0228685

**Published:** 2020-02-03

**Authors:** Naoyuki Kawao, Yoshimasa Takafuji, Masayoshi Ishida, Katsumi Okumoto, Hironobu Morita, Masafumi Muratani, Hiroshi Kaji

**Affiliations:** 1 Department of Physiology and Regenerative Medicine, Kindai University Faculty of Medicine, Osakasayama, Japan; 2 Life Science Research Institute, Kindai University, Osakasayama, Japan; 3 Department of Physiology, Gifu University Graduate School of Medicine, Gifu, Japan; 4 Department of Genome Biology, Faculty of Medicine, University of Tsukuba, Tsukuba, Japan; University of Melbourne, AUSTRALIA

## Abstract

The vestibular system controls balance, posture, blood pressure, and gaze. However, the roles of the vestibular system in energy and glucose metabolism remain unknown. We herein examined the roles of the vestibular system in obesity and impaired glucose metabolism using mice with vestibular lesions (VL) fed a high-sucrose/high-fat diet (HSHFD). VL was induced by surgery or arsenic. VL significantly suppressed body fat enhanced by HSHFD in mice. Glucose intolerance was improved by VL in mice fed HSHFD. VL blunted the levels of adipogenic factors and pro-inflammatory adipokines elevated by HSHFD in the epididymal white adipose tissue of mice. A β-blocker antagonized body fat and glucose intolerance enhanced by HSHFD in mice. The results of an RNA sequencing analysis showed that HSHFD induced alterations in genes, such as insulin-like growth factor-2 and glial fibrillary acidic protein, in the vestibular nuclei of mice through the vestibular system. In conclusion, we herein demonstrated that the dysregulation of the vestibular system influences an obese state and impaired glucose metabolism induced by HSHFD in mice. The vestibular system may contribute to the regulation of set points under excess energy conditions.

## Introduction

The linear and angular acceleration of the head is sensed by vestibular epithelial cells, and then transmitted to vestibular nuclei through vestibular neurons. The neurons of vestibular nuclei are the control center of balance perception, and the vestibular system is connected to other neuronal tracts, such as the vestibulo-oculomotor and vestibulo-spinal tracts, which regulate eye gaze by vestibulo-ocular reflexes and posture, respectively [1,2]. Moreover, vestibular nucleus neurons connect to the autonomic nervous system and regulate the cardiovascular system as vestibulo-autonomic reflexes [2,3].

Regarding the relationships between the vestibular system and skeletal organs, previous studies revealed that vestibular lesions (VL) decrease bone mineral density (BMD) through the sympathetic nervous system in rodents [4,5]. Luxa et al. reported that labyrinthectomy increased myofiber remodeling in the soleus muscle of mice [6]. We recently revealed that gravity changes affect muscle and bone through the vestibular system in mice [7,8]. Collectively, these findings suggest that the vestibular system regulates the musculoskeletal system partly through the sympathetic nervous system. Dysfunctions in the vestibular system clinically cause dizziness, vertigo, and unsteadiness [9]. Long-term space flight impairs the vestibular system in astronauts who experience orthostatic intolerance and unsteadiness [3]. However, the roles of the vestibular system in metabolic homeostasis have not yet been elucidated in detail.

Excess energy is stored in white adipose tissue (WAT) as lipids and causes obesity, a risk factor for diabetes [10]. Adipocytes differentiate from mesenchymal stem cells, and the activation of adipogenic differentiation is followed by enhanced levels of peroxisome proliferator-activated receptor γ (PPARγ), aP2, long chain acyl-CoA synthetase (ACSL) 1, and lipoprotein lipase (LPL). In obesity, WAT releases pro-inflammatory adipokines, such as tumor necrosis factor (TNF)-α, plasminogen activator inhibitor (PAI)-1, and monocyte chemoattractant protein (MCP)-1, which impair glucose metabolism due to the induction of low-grade systemic inflammation [10]. Adiponectin and leptin released from adipose tissue exert pleiotropic effects in glucose metabolism [10]. Circulating adiponectin and leptin produced from WAT are negatively and positively related to fat mass, respectively [11,12]. Previous findings showed that the activated hypothalamic leptin/melanocortin system, decreased adiponectin levels, and hyperinsulinemia enhance sympathetic nervous activity in obesity [13–15].

The regulation of energy and glucose metabolism by the autonomic nervous system has been well established. Sympathetic β agonists directly stimulate glycogenolysis and gluconeogenesis mainly through β_2_ receptors [16]. Catecholamines stimulate lipolysis through β_1_ and β_2_ adrenergic receptors [17]. β_3_ adrenergic receptors mediate lipolysis and glucose uptake in adipocytes [18]. Since chronic hyperglycemia in diabetes causes peripheral neuropathy, particularly in sensory nerves and the autonomic nervous system, auditory and vestibular dysfunctions are often comorbid with diabetes [19]. Long-term space flight increases insulin resistance and impairs the vestibular function in astronauts [3,20]. Several centrifugation studies suggest that the macular gravity receptor (MGR) influences the Medial Vestibular Nucleus (MVe) and further projects into many of the homeostatic nuclei of the brain stem and hypothalamus that are responsible for energy regulation and metabolic homeostasis [21]. Fuller et al. reported that wild-type mice subjected to vestibular stimulation via centrifugation for a period of 8 weeks exhibit an initial decrease in food intake and prolonged body fat reduction, which are not observed in macular otoconia-deficient mice [22]. These findings suggest that the vestibular system influences metabolism and energy regulation [21,22]. However, the details in the mechanisms by which the vestibular system regulates energy and glucose metabolism remain unclear.

In the present study, we examined the influence and mechanism of action of VL on obesity and impaired glucose metabolism using high-sucrose/high-fat diet (HSHFD)-fed obese mice with VL induced by surgery and toxic chemicals to clarify the roles of the vestibular system in diet-induced obesity and impaired glucose metabolism.

## Materials and methods

### Ethics statement

All animal experiments were performed in accordance with the guidelines of the National Institutes of Health and the institutional rules for the use and care of laboratory animals at Kindai University. All procedures were approved by the Experimental Animal Welfare Committee of Kindai University (Permit number: KAME-27-029). All efforts were made to minimize suffering. Mice were euthanized with excess isoflurane.

### Animal experiments

Male C57BL/6J mice were purchased from CLEA Japan (Tokyo, Japan). Mice were fed *ad libitum* with HSHFD (28% of calories from carbohydrates and 55% from fat, Oriental Yeast, Tokyo, Japan) or a normal diet (ND) and water from 9 weeks old for 4 or 8 weeks. After 6 hours of fasting, mice were euthanized with excess isoflurane and tissue samples were collected.

### Surgical vestibular lesions (sVL)

Male C57BL/6J mice (7 weeks old) were randomly divided into 4 groups: ND/Sham (n = 8), ND/sVL (n = 8), HSHFD/Sham (n = 8), and HSHFD/sVL (n = 8). VL surgery was bilaterally performed in accordance with the method described previously in mice under 2% isoflurane anesthesia [7]. Briefly, ear ossicles were removed through the external auditory canal. The vestibule was lesioned by inserting a dental reamer through the oval window in the inner ear followed by ablation using a cautery apparatus. The effects of VL surgery on the vestibular system were assessed by a swimming test, as described previously [23], in which mice with vestibular dysfunction could not swim and continue to turn under the warm water (35°C). In sham mice, the tympanic membrane was removed, but ear ossicles and vestibule remained. After a 2-week recovery period, mice were fed ND or HSHFD for 8 weeks.

### Arsenic-induced vestibular lesions (aVL)

Male C57BL/6J mice (9 weeks old) were randomly divided into 4 groups: ND/Control (n = 8), ND/aVL (n = 8), HSHFD/Control (n = 8), and HSHFD/aVL (n = 8). VL using sodium arsanilate was bilaterally induced in accordance with the previously described method [24]. Under 2% isoflurane anesthesia, 10 μl sodium arsanilate (Tokyo Chemical Industry, Tokyo, Japan) dissolved in saline was injected into the middle ear cavity (1.5 mg/ear). In control mice, same volume of saline was bilaterally injected. Mice were fed ND or HSHFD for 4 weeks from 1 day after the procedure for aVL.

### Propranolol treatment

Male C57BL/6J mice were divided into 4 groups: ND/Control (n = 8), ND/propranolol (n = 8), HSHFD/Control (n = 8), and HSHFD/propranolol (n = 8). Propranolol (Sigma, St. Louis, MO, USA) was administered to 9-week-old mice via drinking water at 0.5 g/l for 8 weeks, as described previously [8].

### Glucose and insulin tolerance tests

Glucose (Wako, Osaka, Japan) at 1.5 g/kg and insulin (Eli Lilly Japan, Kobe, Japan) at 0.5 U/kg were administered intraperitoneally to mice for glucose and insulin tolerance tests, respectively. Blood glucose levels were measured before and 30, 60, 90, and 120 minutes after the injection.

### Measurement of grip strength

The grip strength of mice was measured five times using a grip strength meter (1027SM, Columbus Instruments, Columbus, OH, USA) and the results obtained were expressed as an average, as described previously [25].

### Quantitative computed tomography (QCT)

After mice were anesthetized with 2% isoflurane, a QCT scan was performed using an X-ray CT system (Latheta LCT-200; Hitachi Aloka Medical, Tokyo, Japan) with the following parameters: a 500-μA tube current, 50-kVp tube voltage, and 48-mm axial field of view, as described previously [7]. In analyses of total fat and muscle masses as well as total bone mineral content (BMC), CT images (voxel size of 96×192×1008 μm) were acquired, and the region of interest was defined as the whole body. In analyses of tibial trabecular and cortical BMD, CT images with a 24-μm isotropic voxel size were obtained, and regions of interest were defined as 1680-μm segments from 96 μm distal to the end of the proximal growth plate towards the diaphysis and 2160-μm segments of the mid-diaphysis, respectively. CT images were analyzed using LaTheta software (version 3.41).

### Histological analysis

Epididymal WAT was fixed in 4% paraformaldehyde for 24 hours, embedded in paraffin, and cut into 4-μm-thick sections. Sections were stained with hematoxylin and eosin, and photographed using a microscope (E800; Canon, Tokyo, Japan) with a CCD camera. Cross-sectional areas of at least 350 adipocytes were quantified by planimetry using ImageJ in a blinded manner.

### Real-time PCR analysis

Total RNA was extracted using the RNeasy Mini kit (Qiagen, Hilden, Germany) in accordance with the manufacturer’s instructions. cDNA was synthesized using a high capacity cDNA reverse transcription kit (Thermo Fisher Scientific, Waltham, MA, USA). A real-time PCR analysis was performed using an ABI PRISM 7900HT (Thermo Fisher Scientific) and Fast SYBR Green Master Mix (Thermo Fisher Scientific), as described previously [25]. Primers used for real-time PCR were shown in S1 Table. The relative mRNA levels of target genes were analyzed by the ΔΔCt method and normalized with 18S rRNA levels.

### Blood chemistry

Serum insulin, leptin, and adiponectin levels were assessed using a mouse insulin enzyme-linked immunosorbent assay kit (Cat. No. AKRIN-011T, FUJIFILM Wako Shibayagi, Gunma, Japan), mouse/rat leptin Quantikine ELISA kit (Cat. No. MOB00, R&D systems, Minneapolis, MN, USA), and mouse/rat adiponectin enzyme-linked immunosorbent assay kit (Cat. No. AKMAN011, FUJIFILM Wako Shibayagi), respectively.

### RNA sequencing

One-millimeter-thick coronal slices were obtained from mouse brains using a mouse brain matrix (Muromachi Kikai, Tokyo, Japan). Vestibular nuclei were collected using a punch according to the Allen Mouse Brain Atlas [26]. Total RNA was extracted from biopsy samples using TRIzol reagent (Thermo Fisher Scientific) and assessed using an Agilent Bioanalyzer with the RNA 6000 Pico Kit (Agilent, Santa Clara, CA, USA). rRNA depletion and library synthesis were performed using the NEBNext rRNA Depletion Kit (E6310, New England Biolabs, Ipswich, MA, USA) and NEBNext Ultra Directional RNA Library Prep Kit (E7420, New England Biolabs) from 500 ng of total RNA. The quality of the library was checked using the Agilent Bioanalyzer with the DNA High-sensitivity kit (Cat. No. 5067–4626, Agilent). Each library was sequenced using Illumina (2×36-bp paired-end reads) with NextSeq500 High Output Kit v2 (Illumina, San Diego, CA, USA). FASTQ files were imported to CLC Genomics Workbench (ver.10.1.1, Qiagen, Germantown, MD, USA). Reads were mapped to mm10 mouse reference genome and quantified for 49,585 annotated genes. Reads per kilobase of transcript per million mapped reads (RPKM) values were normalized by quantile method.

### Statistical analysis

Data are expressed as means ± standard errors of the mean. Significant differences were analyzed using a two-way analysis of variance followed by the Tukey-Kramer test. P values of less than 0.05 were considered to be significant. Statistical analyses were performed using GraphPad PRISM 7.00 (GraphPad Software, San Diego, CA, USA).

## Results

### Effects of sVL on HSHFD-induced obesity

**Fig 1:**
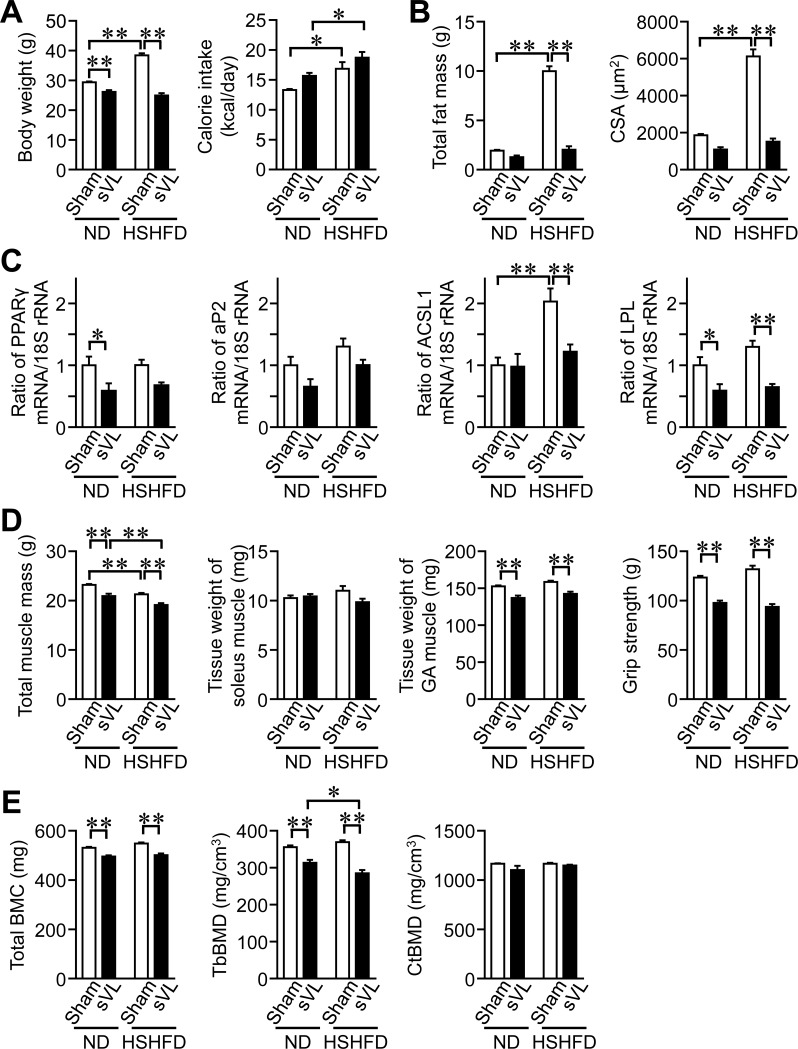
Effects of sVL on body weight and composition in mice fed HSHFD for 8 weeks. (A) Data on body weight and calorie intake from sham surgery and sVL mice fed ND or HSHFD. Body weight was measured 8 weeks after ND or HSHFD feeding. Food intake was collected for 3 days on days 54 to 56 after ND or HSHFD feeding was started and shown as a representative of the average daily calorie intake. (B) Fat mass in the whole body of sham surgery and sVL mice was assessed by QCT 8 weeks after ND or HSHFD feeding was started. The cross-sectional area (CSA) of adipocytes in the white epididymal adipose tissue (WAT) of sham surgery and sVL mice 8 weeks after ND or HSHFD feeding was started. (C) Total RNA was extracted from the epididymal WAT of sham surgery and sVL mice 8 weeks after ND or HSHFD feeding was started. A real-time PCR analysis was then performed. Data are expressed relative to the levels of 18S rRNA. (D) Muscle mass in the whole body of sham surgery and sVL mice was assessed by QCT 8 weeks after ND or HSHFD feeding was started. The tissue weights of the soleus and gastrocnemius (GA) muscles were measured 8 weeks after ND or HSHFD feeding. The grip strengths of the four limbs were measured by a grip strength meter in sham surgery and sVL mice 8 weeks after ND or HSHFD feeding was started. (E) Total BMC, trabecular (Tb) BMD, and cortical (Ct) BMD in the tibia of sham surgery and sVL mice were assessed by QCT 8 weeks after ND or HSHFD feeding was started. *P < 0.05 and **P < 0.01 (Tukey-Kramer test). Data represent the mean ± SEM of 8 mice in each group.

Body weight, total fat mass, and adipocyte size in epididymal WAT were significantly higher in HSHFD-fed mice than in ND-fed mice (Fig 1A and 1B). sVL significantly reduced body weight, total fat mass, and adipocyte size enhanced by HSHFD, but did not affect calorie intake with or without HSHFD (Fig 1A and 1B). sVL suppressed ACSL1 mRNA levels enhanced by HSHFD in the epididymal WAT of mice (Fig 1C), while HSHFD did not affect the mRNA levels of PPARγ, aP2, or LPL in the epididymal WAT of mice (Fig 1C). Total muscle mass was significantly lower in HSHFD-fed mice, and sVL significantly reduced total muscle mass with or without HSHFD (Fig 1D). Although HSHFD feeding did not affect the tissue weights of the soleus and gastrocnemius muscles or grip strength in mice, sVL significantly reduced the tissue weight of the gastrocnemius muscle in ND- or HSHFD-fed mice (Fig 1D). Regarding bone, HSHFD feeding for 8 weeks did not affect total BMC or tibial trabecular and cortical BMD in mice (Fig 1E). sVL significantly decreased total BMC and tibial trabecular BMD in mice fed ND and HSHFD (Fig 1E).

### Effects of sVL on glucose metabolism in mice fed HSHFD

**Fig 2:**
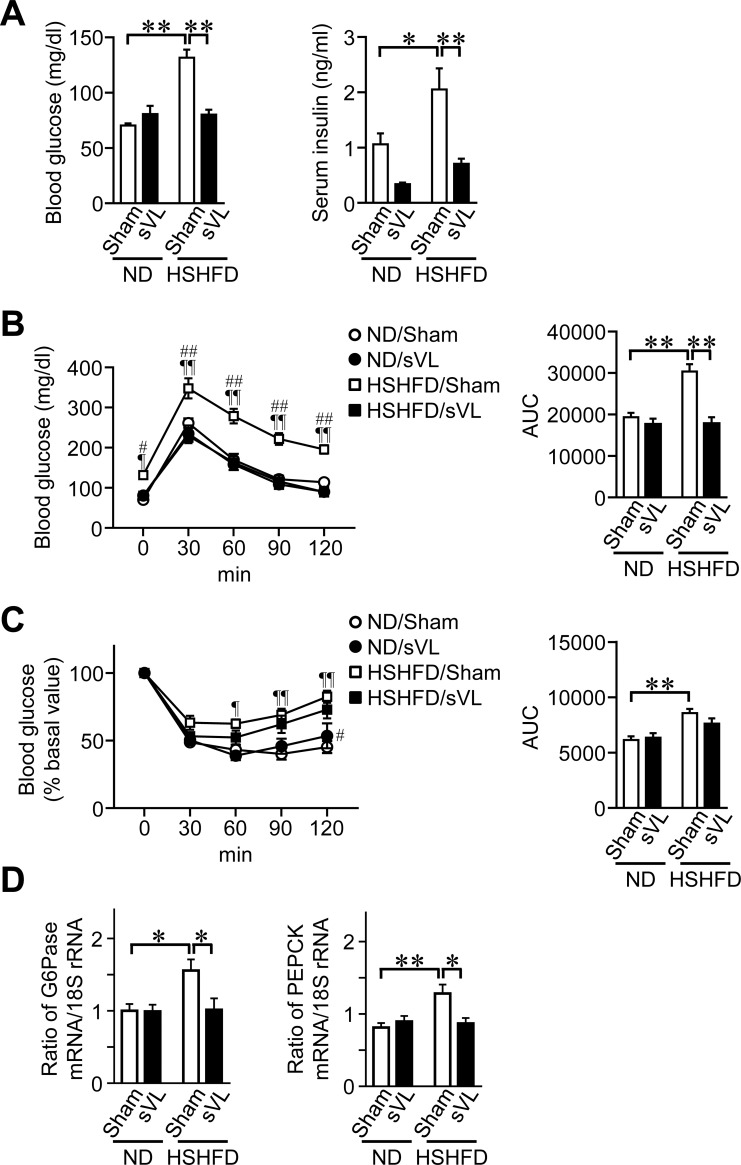
Effects of sVL on glucose metabolism in mice fed HSHFD for 8 weeks. (A) Fasting blood glucose and serum insulin levels were measured 8 weeks after ND or HSHFD feeding was started. (B, C) Responses of blood glucose to a single intraperitoneal injection of glucose (B) and insulin (C) in sham surgery and sVL mice 8 weeks after ND or HSHFD feeding was started. The area under the curve (AUC) for 120 min was calculated. (D) Total RNA was extracted from liver tissues of sham surgery and sVL mice 8 weeks after ND or HSHFD feeding was started. A real-time PCR analysis was then performed. Data are expressed relative to 18S rRNA levels. *P < 0.05 and **P < 0.01; ¶*P* < 0.05 and ¶¶*P* < 0.01, vs ND/Sham; #*P* < 0.05 and ##*P* < 0.01, vs HSHFD/sVL (Tukey-Kramer test). Data represent the mean ± SEM of 8 mice in each group.

The levels of fasting blood glucose and serum insulin were significantly higher in mice fed HSHFD than in mice fed ND (Fig 2A). sVL significantly reduced the levels of fasting blood glucose and serum insulin elevated by HSHFD feeding for 8 weeks in mice (Fig 2A). sVL improved glucose intolerance, but not insulin resistance, in mice fed HSHFD (Fig 2B and 2C). sVL significantly suppressed the mRNA levels of key enzymes of gluconeogenesis such as glucose-6-phosphatase (G6Pase) and phosphoenolpyruvate carboxykinase (PEPCK) enhanced by HSHFD in mouse liver tissues (Fig 2D).

### Effects of aVL on obesity and impaired glucose metabolism in HSHFD-fed mice

**Fig 3:**
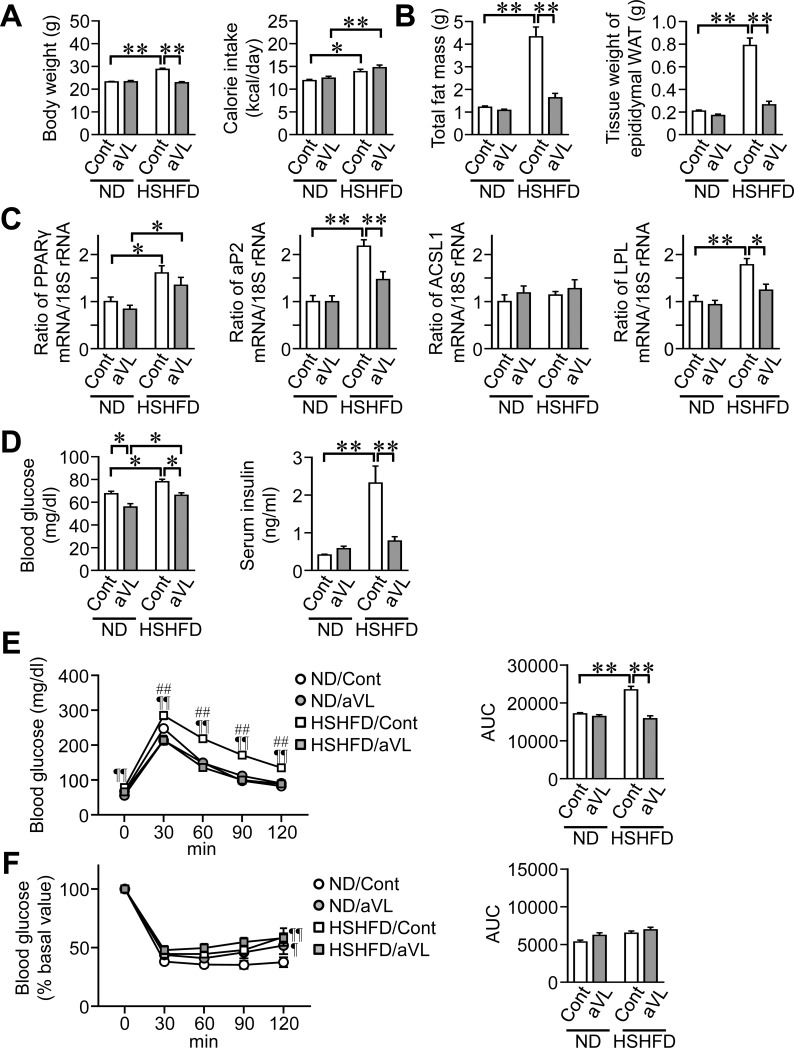
Effects of aVL on fat mass and glucose metabolism in mice fed HSHFD for 4 weeks. (A) Data on body weight and calorie intake from control (Cont) and aVL mice fed ND or HSHFD. Body weight was measured 4 weeks after ND or HSHFD feeding. Food intake was collected for 3 days on days 26 to 28 after ND or HSHFD feeding was started and shown as a representative of the average daily calorie intake. (B) Fat mass in the whole body of control and aVL mice was assessed by QCT 4 weeks after ND or HSHFD feeding was started. The tissue weight of epididymal white adipose tissue (WAT) was measured 4 weeks after ND or HSHFD feeding. (C) Total RNA was extracted from the epididymal WAT of control and aVL mice 4 weeks after ND or HSHFD feeding was started. A real-time PCR analysis was then performed. Data are expressed relative to the levels of 18S rRNA. (D) Fasting blood glucose and insulin levels were measured 4 weeks after ND or HSHFD feeding was started. (E, F) Responses of blood glucose to a single intraperitoneal injection of glucose (E) and insulin (F) in control and aVL mice 4 weeks after ND or HSHFD feeding was started. The area under the curve (AUC) for 120 min was calculated. *P < 0.05 and **P < 0.01; ¶*P* < 0.05 and ¶¶*P* < 0.01, vs ND/Sham; ##*P* < 0.01, vs HSHFD/aVL (Tukey-Kramer test). Data represent the mean ± SEM of 8 mice in each group.

We examined the effects of aVL on obesity and impaired glucose metabolism in mice fed HSHFD for 4 weeks because the vestibular function was recovered 8 weeks after aVL. Body weight, total fat mass, and the tissue weight of epididymal WAT were higher in mice fed HSHFD than in those fed ND for 4 weeks (Fig 3A and 3B). aVL significantly reduced body weight, total fat mass, and the tissue weight of epididymal WAT enhanced by HSHFD feeding (Fig 3A and 3B). Although the mRNA levels of PPARγ, aP2, and LPL were higher in mice fed HSHFD for 4 weeks than in those fed ND, aVL significantly decreased the mRNA levels of aP2 and LPL enhanced by HSHFD (Fig 3C). aVL reduced fasting blood glucose levels in mice fed ND and HSHFD, and significantly suppressed serum insulin levels enhanced by HSHFD feeding in mice (Fig 3D). Although HSHFD feeding for 4 weeks induced glucose intolerance, but not insulin resistance, aVL improved glucose intolerance in mice fed HSHFD (Fig 3E and 3F).

### Effects of VL on adipokine levels in mice fed HSHFD

**Fig 4:**
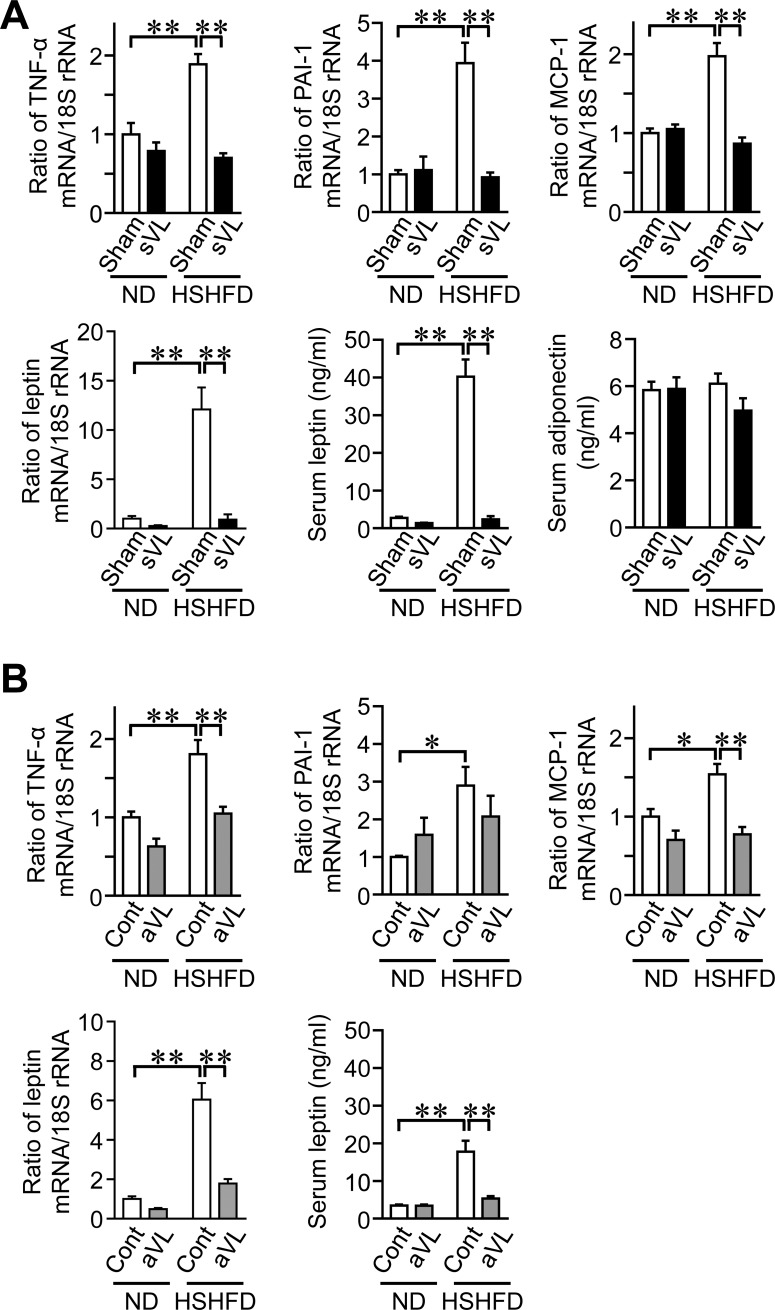
Effects of sVL and aVL on adipokine levels in mice fed HSHFD. (A) Total RNA was extracted from the epididymal WAT of sham surgery and sVL mice 8 weeks after ND or HSHFD feeding was started. A real-time PCR analysis was then performed. Data are expressed relative to 18S rRNA levels. Serum samples were collected from sham surgery and sVL mice 8 weeks after ND and HSHFD feeding was started. The quantification of serum leptin and adiponectin levels was performed. (B) Total RNA was extracted from the epididymal WAT of control (Cont) and aVL mice 4 weeks after ND or HSHFD feeding was started. A real-time PCR analysis was then performed. Data are expressed relative to the levels of 18S rRNA. Serum samples were collected from control and aVL mice 8 weeks after ND and HSHFD feeding was started. The quantification of serum leptin levels was performed. *P < 0.05 and **P < 0.01 (Tukey-Kramer test). Data represent the mean ± SEM of 8 mice in each group.

sVL significantly suppressed the mRNA levels of TNF-α, PAI-1, MCP-1, and leptin as well as serum leptin levels enhanced by HSHFD feeding for 8 weeks (Fig 4A), while HSHFD feeding for 8 weeks and sVL did not affect serum adiponectin levels in mice (Fig 4A). aVL significantly blunted the mRNA levels of TNF-α, MCP-1, and leptin as well as serum leptin levels enhanced by HSHFD feeding for 4 weeks in mice (Fig 4B).

### Effects of propranolol on obesity, adipokine levels, and glucose intolerance in mice fed HSHFD

**Fig 5:**
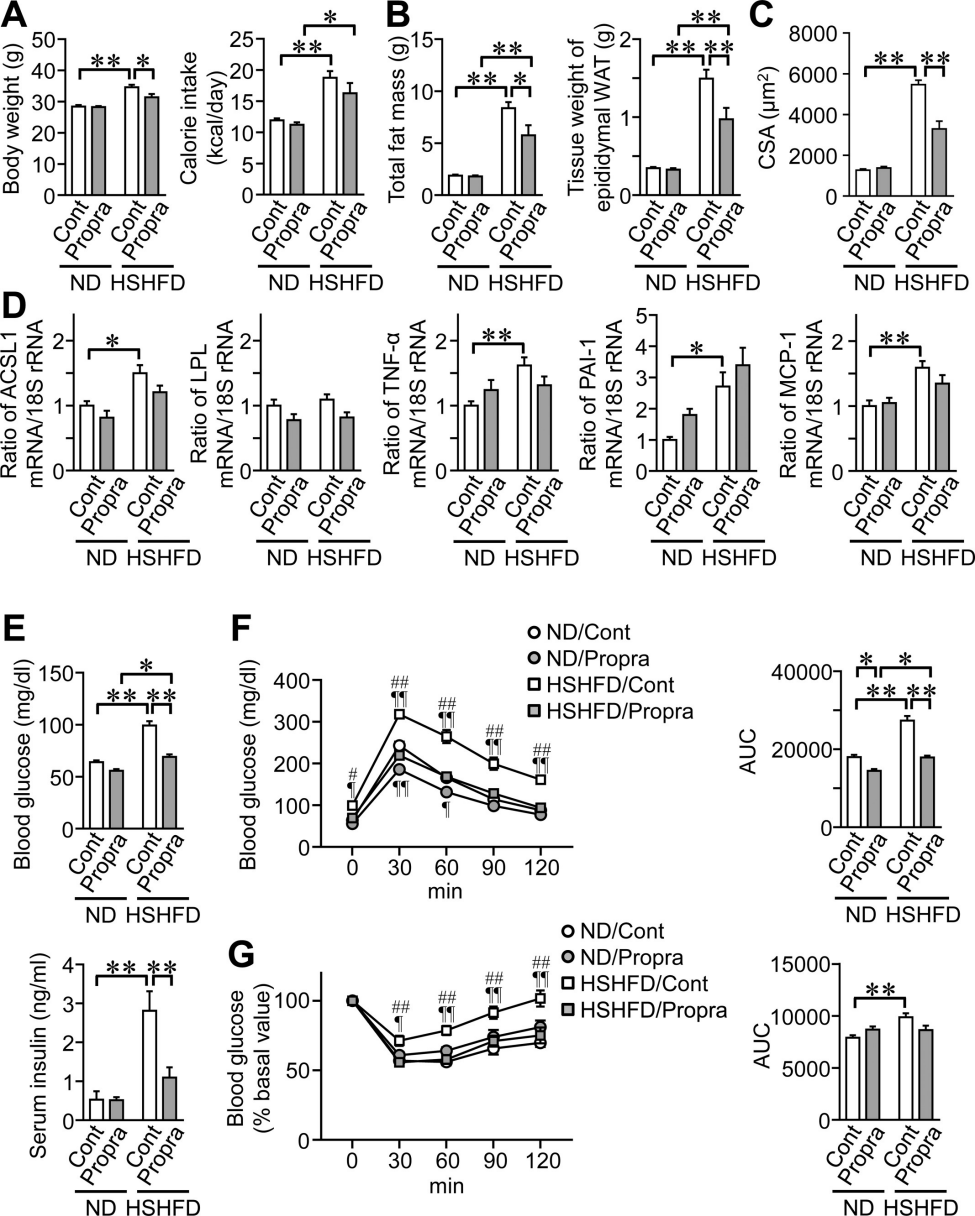
Effects of the propranolol treatment on fat mass and glucose metabolism in mice fed HSHFD for 8 weeks. (A) Data on body weight and calorie intake from mice with or without propranolol (Propra). Body weight was measured 8 weeks after ND or HSHFD feeding. Food intake was collected for 3 days on days 54 to 56 after ND or HSHFD feeding was started and shown as a representative of the average daily calorie intake. (B) Fat mass in the whole body of mice with or without propranolol was assessed by QCT 8 weeks after ND or HSHFD feeding was started. The tissue weight of epididymal WAT was measured 8 weeks after ND or HSHFD feeding. (C) The cross-sectional area (CSA) of adipocytes in the epididymal WAT of mice with or without propranolol 8 weeks after ND or HSHFD feeding was started. (D) Total RNA was extracted from the epididymal WAT of mice with or without propranolol 8 weeks after ND or HSHFD feeding was started. A real-time PCR analysis was then performed. Data are expressed relative to the levels of 18S rRNA. (E) Fasting blood glucose and serum insulin levels were measured 8 weeks after ND or HSHFD feeding was started. (F, G) Responses of blood glucose to a single intraperitoneal injection of glucose (F) and insulin (G) in mice with or without propranolol 8 weeks after ND or HSHFD feeding was started. The area under the curve (AUC) for 120 min was calculated. Cont; control. *P < 0.05 and **P < 0.01; ¶*P* < 0.05 and ¶¶*P* < 0.01, vs ND/Control; #*P* < 0.05 and ##*P* < 0.01, vs HSHFD/Propranolol (Tukey-Kramer test). Data represent the mean ± SEM of 8 mice in each group.

The vestibular system contributes to the control of the cardiovascular and skeletal systems through the sympathetic nervous system. We therefore examined the role of the sympathetic nervous system in HSHFD-fed mice using the β-blocker, propranolol. Propranolol significantly reduced body weight, total fat mass, the tissue weight of epididymal WAT, and adipocyte sizes enhanced by HSHFD feeding for 8 weeks in mice (Fig 5A–5C). Propranolol did not affect the mRNA levels of ACSL1, TNF-α, PAI-1, or MCP-1 enhanced by HSHFD feeding in mice (Fig 5D). Propranolol significantly reduced fasting blood glucose and serum insulin levels increased by HSHFD feeding in mice (Fig 5E). Propranolol improved glucose intolerance and seemed to improve insulin resistance in mice fed HSHFD, although its effects on insulin resistance were not significant (Fig 5F and 5G).

### Effects of HSHFD feeding and sVL on gene expression in vestibular nuclei in mice

**Fig 6:**
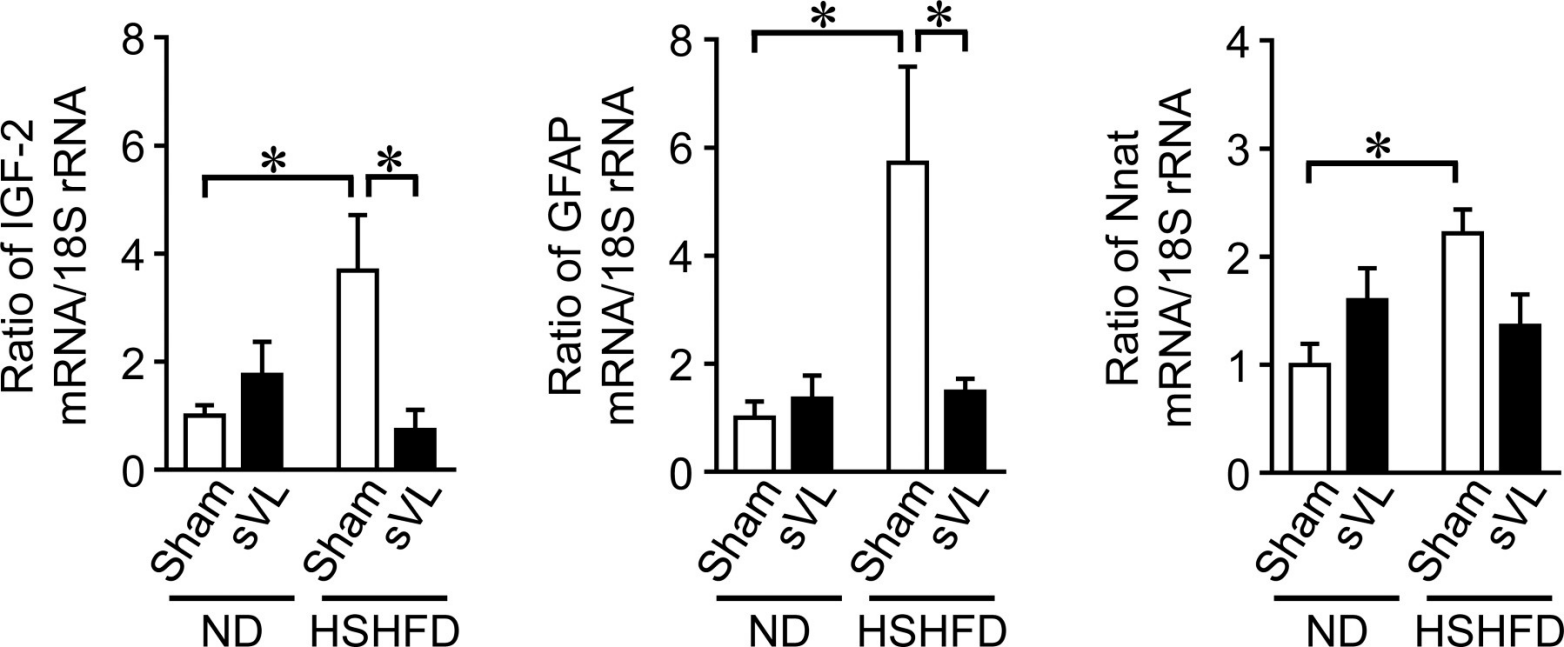
Effects of sVL and HSHFD feeding on gene expression in vestibular nuclei of mice. Total RNA was extracted from the vestibular nuclei of sham surgery and sVL mice 8 weeks after ND or HSHFD feeding was started. A real-time PCR analysis was then performed. Data are expressed relative to the levels of 18S rRNA. *P < 0.05 (Tukey-Kramer test). Data represent the mean ± SEM of 6 mice in each group.

Since the vestibular nuclei is considered to be the center in central nervous system of the vestibular system leading to peripheral vestibular oculomotor, motor and sympathetic nerve systems, we speculated that some factors expressed in the vestibular nuclei might be important for the regulation of the vestibular system on energy metabolism. We therefore performed RNA sequencing analyses on vestibular nuclei between ND- and HSHFD-fed mice with or without sVL to examine the effects of HSHFD feeding for 8 weeks and sVL on gene expression in the vestibular nuclei of mice. The levels of 25 gene transcripts were 2-fold higher in HSHFD/Sham mice than in ND/Sham mice and 0.5-fold lower in HSHFD/sVL mice than in HSHFD/Sham mice (S2 Table). HSHFD for 8 weeks significantly elevated the mRNA levels of insulin-like growth factor-2 (IGF-2), glial fibrillary acidic protein (GFAP), and Nnat in the vestibular nuclei of mice, while sVL significantly suppressed the mRNA levels of IGF-2 and GFAP enhanced by HSHFD feeding (Fig 6).

## Discussion

In the present study, VL blunted body fat and adipokine expression as well as glucose intolerance enhanced by HSHFD feeding in mice. The adrenergic β-blocker propranolol improved fat mass and glucose intolerance enhanced by HSHFD feeding in mice. Vestibular lesions modulated the expression of several genes, such as IGF-2 and GFAP, enhanced by HSHFD feeding in the vestibular nuclei of mice.

The brain and nervous system are the key regulators of energy homeostasis [27,28]. Previous studies suggested that hypergravity with a centrifuge reduces body fat partly through the vestibulo-hypothalamic pathway in mice [22,29]. Moreover, Abe et al. reported that vestibular system-linked serotonergic neurons are crucial for hypophagia induced by hypergravity in rats [30]. In the present study, we revealed that surgical or toxic chemical-induced VL both blunted total fat mass, the tissue weight of epididymal WAT, and adipocyte size increased by HSHFD feeding in mice. Moreover, VL reduced the expression of adipogenic factors, such as ACSL1, aP2, and LPL, elevated by HSHFD feeding in the epididymal WAT of mice. Collectively, the present results suggest that the vestibular system dysregulation influences body fat elevated by HSHFD in mice. Homeostasis is maintained in physiological and pathological states through the optimal use of energy regulated by various organs, such as the nervous system [31,32]. The vestibular system may be related to the centers that integrate multiple inputs for the regulation of a set-point for body fat.

A chronic energy excess causes obesity accompanied by insulin resistance and hyperinsulinemia [10]. In the present study, VL improved hyperglycemia, hyperinsulinemia, glucose intolerance and the liver expressions of G6Pase and PEPCK induced by HSHFD feeding in mice, although surgical VL did not affect insulin resistance induced by HSHFD feeding in mice. These results suggest that the dysregulation of the vestibular system might influence glucose metabolism partly through the regulation of gluconeogenesis in mice. Alternatively, a review by Sailesh et al. proposed that vestibular stimulation affects diabetic state by increasing insulin secretion through modulating autonomic nerve activity based on some preliminary evidence [33]. Moreover, the vestibular system might affect glucose and energy metabolism through various endocrine factors, including cortisol and thyroid hormone [21]. Since VL significantly suppressed serum insulin levels elevated by HSHFD and the slight, but not significant, effects of VL on blood glucose response abnormality to insulin induced by HSHFD were observed in insulin tolerance tests in our study, the vestibular system might affect glucose metabolism through the complex regulation of insulin secretion and resistance or the mechanisms other than insulin action, such as the regulation of glucagon, catecholamines, growth hormone, glucocorticoids, incretins, thyroid hormone, adipokines and myokines. Elevations in circulating pro-inflammatory adipokines, such as TNF-α, PAI-1, and MCP-1, contribute to low-grade systemic inflammation and enhanced insulin resistance in obesity [10]. We revealed that VL suppressed the expression of pro-inflammatory cytokines enhanced by HSHFD feeding in the epididymal WAT of mice; however, they did not affect serum adiponectin levels with or without HSHFD feeding. These results suggest that the dysregulation of the vestibular system may influence impaired glucose metabolism through decreases in pro-inflammatory adipokine levels in mice fed HSHFD. In the present study, VL antagonized serum leptin levels and leptin mRNA expression in WAT enhanced by HSHFD feeding in mice. Leptin changes induced by the vestibular system may affect pro-opiomelanocortin neurons and agouti-related peptide/neuropeptide Y neurons in the hypothalamus, which control appetite and energy expenditure [27]; however, VL did not affect calorie intake with or without HSHFD in the present study. Further studies are needed to clarify the mechanical insights of the VL effects on glucose metabolism.

Plastic changes in the vestibular system induced by long-term exposure to microgravity have been shown to impair vestibulo-cardiovascular reflexes through the sympathetic nerves in astronauts [3]. Previous studies revealed that VL reduced BMD through the activation of the sympathetic nervous system in rodents [5,34]. Moreover, we reported that an adrenergic β-blocker antagonized the effects of hypergravity on skeletal muscle masses through the vestibular system in mice [8]. These findings suggest that the sympathetic nervous system is involved in the effects of the vestibular system on the cardiovascular system, bone metabolism, and skeletal muscle. In the present study, the adrenergic β-blocker, propranolol, partially blunted increases in total fat mass and adipocyte size by HSHFD feeding in mice, which is consistent with previous findings [35]. Moreover, the β-blocker improved glucose intolerance induced by HSHFD feeding in mice. These results suggest that the sympathetic nervous system is involved in obesity and impaired glucose metabolism induced by HSHFD feeding in mice. On the other hand, the β-blocker did not affect the expression of pro-inflammatory adipokines or adipogenic factors in adipose tissue. Since the adrenergic signal facilitates lipolysis and gluconeogenesis and also inhibits insulin release [16], sympathetic nerves may play some roles in the effects of the vestibular system on lipolysis and glucose metabolism rather than pro-inflammatory cytokine production and adipogenic differentiation. Propranolol is a non-specific β-blocker and it crosses the blood-brain barrier. Thus, effects of propranolol may be multifactorial, including central nervous system and peripheral. Further studies using some more specific sympathetic β_2_ antagonist without crossing the blood-brain barrier or adrenergic β_2_ receptor-deleted mice might be useful to exactly evaluate the contribution of the sympathetic nerve system on VL effects on glucose and energy metabolism.

Our comparative RNA sequence data revealed that HSHFD feeding affects the expression of several genes, such as IGF-2, GFAP, and Nnat, in the vestibular nuclei of mice. IGF-2 is strongly expressed in the central nervous system (CNS) and exerts various functions in brain development, neurological disorders, and metabolic diseases [36,37]. GFAP, a cytoskeleton protein, is abundantly expressed in astrocytes and contributes to several cellular processes, including migration, proliferation, vesicle trafficking, autophagy, and astrocyte-neuron interactions [38]. Nnat, which encodes neuronatin, is expressed in the CNS, pancreatic β cells, and adipocytes, and neuronatin contributes to the development of the CNS and the pathological states of several diseases, such as cancer, obesity, and diabetes [39]. Since VL blunted the expression of IGF-2 and GFAP enhanced by HSHFD feeding in the vestibular nuclei of mice in the present study, IGF-2 and GFAP changes in the vestibular nuclei may be related to changes in energy and glucose metabolism modulated by the vestibular system in the excess energy state in mice. Degerman et al. revealed that components of insulin signaling, including insulin receptors and insulin receptor substrate 1, are expressed in the sensory epithelium of the saccule [40]. Insulin facilitates the proliferation of the vestibular sensory epithelium in rats [41]. These findings suggest that hyperinsulinemia in the obese state influences gene expression in the vestibular nuclei through changes in the vestibular sensory epithelium. Moreover, Xing et al. reported that hyperglycemia induced cochlear hair cell damage through advanced glycation end products and its receptors [42]. Therefore, it may be the case that hyperglycemia also induces damage to vestibular hair cells and alterations in IGF-2, GFAP, and Nnat expression in the vestibular nuclei of mice fed HSHFD. Further studies using brain-specific IGF-2, GFAP or Nnat-deleted mice are necessary to clarify the relationships of those genes and the phenotypes of mice.

Obesity is a risk factor for various disorders, such as cardiovascular diseases, diabetes, and osteoarthritis. Although the prevention and treatment of obesity are important for the extension of health life expectancy in the elderly, effective and non-invasive treatment options for obesity remain limited. A recent study suggested that bariatric surgery is effective for the treatment of obesity partly through changes in the set-point of energy expenditure as well as a decrease in calorie intake [43]. The present study revealed that the vestibular system is involved in the development of obesity and its influences on glucose metabolism induced by energy excess in mice. A previous study showed that a noisy galvanic vestibular stimulation (nGVS), an imperceptible level of GVS, improves balance in patients with bilateral vestibulopathy [44]. Moreover, nGVS ameliorates autonomic nervous and motor functions in patients with central neurodegenerative disorders [45]. Therefore, the vestibular signal modified with nGVS has potential to prevent or treat obesity and impaired glucose metabolism.

In conclusion, we herein provide novel evidence to show that VL prevents high-sucrose/high-fat diet-induced obesity and glucose intolerance partly through the sympathetic nervous in mice. The present results suggest that the vestibular system contributes to the regulation of energy and glucose metabolism under excess energy conditions.

## Supporting information

S1 Table Primers used in real-time PCR experiments. PPARγ, proliferator-activated receptor γ; ACSL1, long chain acyl-CoA synthetase 1; LPL, lipoprotein lipase; G6Pase, glucose-6-phosphatase; PEPCK, phosphoenolpyruvate carboxykinase; TNF-α, tumor necrosis factor-α; PAI-1, plasminogen activator inhibitor-1; MCP-1, monocyte chemoattractant protein-1; IGF-2, insulin like growth factor-2; GFAP, glial fibrillary acidic protein.
(DOCX)

S2 Table Gene transcripts in the vestibular nuclei of mice with HSHFD/Sham versus ND/Sham mice as well as HSHFD/sVL versus HSHFD/Sham mice. The levels of 25 gene transcripts were 2-fold higher in HSHFD/Sham mice than in ND/Sham mice and 0.5-fold lower in HSHFD/sVL mice than in HSHFD/Sham mice.
(DOCX)
