# Supplementary material for: Roles of the vestibular system in obesity and impaired glucose metabolism in high-fat diet-fed mice
Source: PLoS One. 2020 Feb 3;15(2):e0228685. doi: 10.1371/journal.pone.0228685 (PMC6996831; doi:10.1371/journal.pone.0228685)
Supplement: S1 Table — PPARγ, proliferator-activated receptor γ; ACSL1, long chain acyl-CoA synthetase 1; LPL, lipoprotein lipase; G6Pase, glucose-6-phosphatase; PEPCK, phosphoenolpyruvate carboxykinase; TNF-α, tumor necrosis factor-α; PAI-1, plasminogen activator inhibitor-1; MCP-1, monocyte chemoattractant protein-1; IGF-2, insulin like growth factor-2; GFAP, glial fibrillary acidic protein. (DOCX) [file pone.0228685.s001.docx]

**S1 Table.** Primers used in real-time PCR experiments.

| Gene |  | Primer sequence |
| --- | --- | --- |
| PPARγ  aP2  ACSL1  LPL  G6Pase  PEPCK  TNF-α | Forward  Reverse  Forward  Reverse  Forward  Reverse  Forward  Reverse  Forward  Reverse  Forward  Reverse  Forward  Reverse | 5’-GGAAAGACAACGGACAAATCAC-3’  5’-TACGGATCGAAACTGGCAC-3’  5’-ATCACCGCAGACGACAGGA-3’  5’-CTCATGCCCTTTCATAAACT-3’  5’-CGGGCAGCGGAGGAGAATTCT-3’  5’-GGTGCGCACGTACTGCCGAA-3’  5’-GCTGGTGGGAAATGATGTG-3’  5’-TGGACGTTGTCTAGGGGGTA-3’  5’-AGATGGTGTGAGCGGCCAGA-3’  5’-CAACCCCAAGAGGGTTCCCA-3’  5’-GACACAGTGCCCATCCCCAA-3’  5’-TGGGAACCTGGCGTTGAATG-3’  5’-CCCAGACCCTCACACTCAGATC-3’  5’-GCCACTCCAGCTGCTCCTC-3’ |
| PAI-1 | Forward  Reverse | 5’-TTCAGCCCTTGCTTGCCTC-3’  5’-ACACTTTTACTCCGAAGTCGGT-3’ |
| MCP-1 | Forward  Reverse | 5’-CCACTCACCTGCTGCTACTCA-3’  5’-TGGTGATCCTCTTGTAGCTCTCC-3’ |
| Leptin  IGF-2  GFAP  Nnat | Forward  Reverse  Forward  Reverse  Forward  Reverse  Forward  Reverse | 5’-TGACACCAAAACCCTCATCA-3’  5’-AGCCCAGGAATGAAGTCCA-3’  5’-ACAACTTCGATTTGAACCACATTC-3’  5’-GAGAGCTCAAACCATGCAAACT-3’  5’-GCGAAGAAAACCGCATCACC-3’  5’-TGGCAGGGCTCCATTTTCAA-3’  5’-CACCCACTTTCGGAACCATG-3’  5’-GCACGCGGAAGATGTACCAG-3’ |
| 18S rRNA | Forward | 5’-CGGCTACCACATCCAAGGAA-3’ |
|  | Reverse | 5’-GCTGGAATTACCGCGGCT-3’ |

PPARγ, proliferator-activated receptor γ; ACSL1, long chain acyl-CoA synthetase 1; LPL, lipoprotein lipase; G6Pase, glucose-6-phosphatase; PEPCK, phosphoenolpyruvate carboxykinase; TNF-α, tumor necrosis factor-α; PAI-1, plasminogen activator inhibitor-1; MCP-1, monocyte chemoattractant protein-1; IGF-2, insulin like growth factor-2; GFAP, glial fibrillary acidic protein.
