# Supplementary material for: Roles of the vestibular system in obesity and impaired glucose metabolism in high-fat diet-fed mice
Source: PLoS One. 2020 Feb 3;15(2):e0228685. doi: 10.1371/journal.pone.0228685 (PMC6996831; doi:10.1371/journal.pone.0228685)
Supplement: S2 Table — The levels of 25 gene transcripts were 2-fold higher in HSHFD/Sham mice than in ND/Sham mice and 0.5-fold lower in HSHFD/sVL mice than in HSHFD/Sham mice. (DOCX) [file pone.0228685.s002.docx]

**S2 Table** Gene transcripts in the vestibular nuclei of mice with HSHFD/Sham versus ND/Sham mice as well as HSHFD/sVL versus HSHFD/Sham mice.

| Gene | Gene accession number | Fold change | |
| --- | --- | --- | --- |
|  |  | HSHFD/Sham  vs. ND/Sham (≥2.00) | HSHFD/sVL  vs. HSHFD/Sham  (≤0.50) |
| Igf2 | NM_001122736 | 9.58 | 0.14 |
| Gfap | NM_001131020 | 8.26 | 0.40 |
| Gm23787 | - | 4.85 | 0.30 |
| Nnat | NM_010923 | 4.39 | 0.35 |
| Nbl1 | NM_008675 | 4.03 | 0.47 |
| Fn1 | NM_001276408 | 3.98 | 0.23 |
| Ptgds | NM_008963 | 3.87 | 0.18 |
| Plac9b | NM_001270503 | 3.71 | 0.10 |
| Dcn | NM_001190451 | 3.70 | 0.30 |
| Ezr | NM_009510 | 3.46 | 0.41 |
| Ahnak | NM_001039959 | 3.33 | 0.34 |
| Mir323 | NR_029757.1 | 3.23 | 0.34 |
| Thbd | NM_009378 | 3.18 | 0.27 |
| Slc7a11 | NM_011990 | 2.87 | 0.49 |
| Pltp | NM_011125 | 2.86 | 0.49 |
| Col1a1 | NM_007742 | 2.82 | 0.35 |
| Foxj1 | NM_008240 | 2.75 | 0.32 |
| Vtn | NM_011707 | 2.70 | 0.32 |
| Ifitm3 | NM_025378 | 2.61 | 0.29 |
| Acta2 | NM_007392 | 2.46 | 0.40 |
| Syt10 | NM_018803 | 2.42 | 0.46 |
| Zfp36l1 | NM_007564 | 2.41 | 0.39 |
| Dchs1 | NM_001162943 | 2.31 | 0.42 |
| Ptrf | NM_008986 | 2.11 | 0.44 |
| Gm25711 | - | 2.03 | 0.18 |
